# Application of bi-clustering of gene expression data and gene set enrichment analysis methods to identify potentially disease causing nanomaterials

**DOI:** 10.1016/j.dib.2017.10.060

**Published:** 2017-10-26

**Authors:** Andrew Williams, Sabina Halappanavar

**Affiliations:** Environmental Health Science and Research Bureau, Health Canada, Ottawa, Ontario, Canada K1A 0K9

**Keywords:** Nanomaterials, Toxicogenomics, Predictive toxicology, Bi-clustering

## Abstract

This article contains data related to the research article ‘*Application of bi-clustering of gene expression data and gene set enrichment analysis methods to identify potentially disease causing nanomaterials*’ (Williams and Halappanavar, 2015) [1]. The presence of diverse types of nanomaterials (NMs) in commerce has grown significantly in the past decade and as a result, human exposure to these materials in the environment is inevitable. The traditional toxicity testing approaches that are reliant on animals are both time- and cost- intensive; employing which, it is not possible to complete the challenging task of safety assessment of NMs currently on the market in a timely manner. Thus, there is an urgent need for comprehensive understanding of the biological behavior of NMs, and efficient toxicity screening tools that will enable the development of predictive toxicology paradigms suited to rapidly assessing the human health impacts of exposure to NMs. In an effort to predict the long term health impacts of acute exposure to NMs, in Williams and Halappanavar (2015) [1], we applied bi-clustering and gene set enrichment analysis methods to derive essential features of altered lung transcriptome following exposure to NMs that are associated with lung-specific diseases. Several datasets from public microarray repositories describing pulmonary diseases in mouse models following exposure to a variety of substances were examined and functionally related bi-clusters showing similar gene expression profiles were identified. The identified bi-clusters were then used to conduct a gene set enrichment analysis on lung gene expression profiles derived from mice exposed to nano-titanium dioxide, carbon black or carbon nanotubes (nano-TiO2, CB and CNTs) to determine the disease significance of these data-driven gene sets. The results of the analysis correctly identified all NMs to be inflammogenic, and only CB and CNTs as potentially fibrogenic. Here, we elaborate on the details of the statistical methods and algorithms used to derive the disease relevant gene signatures. These details will enable other investigators to use the gene signature in future Gene Set Enrichment Analysis studies involving NMs or as features for clustering and classifying NMs of diverse properties.

**Specifications Table**

**Table:** 

Organism/cell line/tissue	*Mus Musculus/Lung*
Sequencer or array type	*Agilent-028005 SurePrint G3 Mouse GE 8×60K Microarray*
Data format	*Raw: TXT files; normalized data: TXT files*
Experimental factors	*Exposures to a variety of nanomaterials (nano-titanium dioxide, carbon black, carbon nanotubes)*
Experimental features	*Bi-cluster analysis on publically available data obtained from Gene Expression Omnibus (GEO) describing specific lung diseases was conducted to identify functionally related gene sets. DAVID analysis was conducted on each of the gene sets from this analysis to identify functional representation of each gene set. Gene set enrichment analysis was then conducted on nine toxicogenomic gene expression studies examining the toxicity induced by a variety of nanomaterials to determine the disease significance of the altered gene expression profiles following exposure to NMs.*
Sample source location	*Ottawa, Ontario, Canada*
Data accessibility	*National Centre for Biotechnology Information, GEO database Accession:*GSE35193, GSE41041, GSE47000, GSE60801, GSE61366

**Value of the data**

• The results enabled deeper mechanistic understanding of NM-induced lung toxicity.
• The data enabled the development of a database with toxicity fingerprints that are specific to lung diseases.
• Using the statistical tools and algorithms established, it may be possible to predict the toxicities of new NMs that have yet to undergo experimental testing.
• The data was integral in identifying new gene sets associated with lung pathology that were previously not known.
• The gene sets identified could serve as features for clustering and classifying NMs of diverse properties.

## 1 Data description

We anticipate that the importance of toxicogenomics studies in chemical risk assessment will continue to increase in the coming years. However, its success will depend on 1) accurate and prompt reporting of the data, 2) ensuring public availability of the datasets and 3) sharing of the training sets and tools such as those described here. As the public repository of toxicogenomics datasets for individual NMs is populated, and the underlying mechanisms of NM-induced toxicity are revealed, the data can be used for routine categorization of NMs and their prioritization for further testing.

## 2 Experimental design, materials and methods

### 2.1 Experimental design

**Fig. 1:**
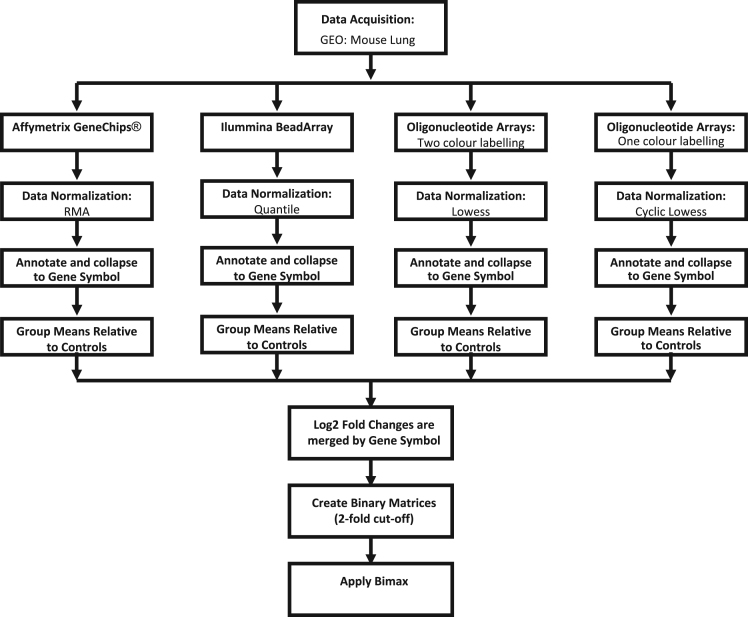
Workflow employed for the discovery phase of the analysis.

The discovery phase was the first step in this study. This involved obtaining publically available data from Gene Expression Omnibus (GEO) describing specific mouse lung diseases. Raw data files were downloaded and processed, normalized and standardized to the control samples. The data from these studies were then merged together and bi-cluster analysis was then conducted. Fig. 1 outlines the various processing steps taken for the gene set discovery phase of the analysis.

DAVID Analysis was then conducted on each of the bi-cluster to interpret the possible functions of the gene set. Gene set enrichment analysis was then conducted using the bi-clusters on a series of toxicogenomics studies on three NMs (nano-TiO2, CB and CNTs) to determine the disease significance of these data-driven gene sets. The specific details of these analyses are outlined below.

### 2.2 Lung disease models

The data used in the discovery phase of novel gene sets relating to lung disease models and lung injury outcomes were obtained from the GEO. The accession numbers for these studies [2], [3], [4], [5], [6], [7], [8], [9], [10], [11], [12], [13] are presented in Table 1. Lung disease models or lung injury outcomes addressed in this analysis included lung inflammation, emphysema, chronic obstructive pulmonary disease, and lung cancer and tumors. These studies utilized several different microarray platforms including the Affymetrix GeneChip®, and Illumina Expression Beadchip.

**Table 1:** Studies used for Gene Set Exploration Phase.

Geo accession	Phenotype/model	Microarray platform (GEO GPL ID)	Reference
GSE4231	Lung inflammation	UCSF 10Mm Mouse v.2 Oligo Array (GPL1089); UCSF GS Operon Mouse v.2 Oligo Array (GPL3330); UCSF 11Mm Mouse v.2 Oligo Array (GPL3331); UCSF 7Mm Mouse v.2 Oligo Array (GPL3359)	[2]
GSE6116	Lung tumors	Affymetrix Mouse Genome 430 2.0 Array (GPL1261)	[3]
GSE6858	Asthma	Affymetrix Mouse Genome 430 2.0 Array (GPL1261)	[4]
GSE8790	Emphysema	Affymetrix Mouse Genome 430 2.0 Array (GPL1261)	[5]
GSE11037	Emphysema	Agilent-011978 Mouse Microarray G4121A (GPL891)	[6]
GSE18534	Small cell lung cancer	Affymetrix Mouse Genome 430 2.0 Array (GPL1261)	[7]
GSE19605	Lung carcinogenesis	Illumina MouseRef-8 v2.0 Expression Beadchip (GPL6885)	[8]
GSE25640	Pulmonary fibrosis	Affymetrix Mouse Genome 430 2.0 Array (GPL1261)	[9]
GSE31013	Spontaneous lung tumors	Affymetrix Mouse Genome 430 2.0 Array (GPL1261)	[10]
GSE40151	Idiopathic pulmonary fibrosis	Affymetrix Mouse Genome 430 2.0 Array (GPL1261)	[11]
GSE42233	Lung cancer	Illumina Mouse WG-6 v2.0 expression beadchip (GPL6887)	[12]
GSE52509	Chronic obstructive pulmonary disease (COPD)	Illumina MouseRef-8 v2.0 Expression Beadchip (GPL6885)	[13]

### 2.3 Data processing and normalization

The log2 transformation was applied to all signal intensity measurements. For the two colour microarray studies, the LOWESS normalization method [14] using the R statistical software environment [15] was applied. For studies using the Affymetrix GeneChips®, the RMA normalization was applied using the justRMA()function in the affy R package [16]. Quantile normalization was applied for studies that utilized the Illumina Expression Beadchip and completed using the lumiN()function in the lumi R package [17].

Probes with technical replicates were averaged using the median; the data for each study was then merged to its appropriate annotation file to obtain the gene symbol and probes with the same gene symbol were averaged using the median. Experimental conditions with biological replicates were also averaged using the median. The average for each of the experimental conditions was normalized to the appropriate control samples resulting in the log2 fold change for each experimental condition. The control samples were then removed from further analysis. All studies were merged together using the gene symbol. The resulting dataset consisted of 8752 gene symbols.

### 2.4 Bi-clustering

The bi-clustering data analysis was conducted in R using the biclust package [18]. The Bimax method [19] was selected for this analysis. Bimax uses a simple data model that assumes two possible states for each expression level, no change and change with respect to a control experiment. For this analysis, two binary matrices were constructed. One matrix representing zero's and one's where the one's indicate genes that were 2-fold up regulated and the second matrix where the one's identify genes that were 2-fold down regulated. These two matrices were analyzed independently.

The option for the minimum number of rows for the Bimax method was set at 15 and the minimum number of columns (which represent the experimental conditions) was set at 5 with the maximum number of columns set at 15. This resulted in 8 bi-clusters from the binary matrix representing the up regulated genes and 2 bi-clusters were identified for the matrix representing the down regulated genes.

### 2.5 DAVID analysis

Gene lists from each bi-cluster were submitted to DAVID (https://david-d.ncifcrf.gov/) for functional annotation [20], [21]. Gene lists were pasted into the web application and the “Official_Gene_Symbol” was selected as the gene identifier. Mus Musculus was selected as the species which was used as the background for the analysis. Default settings were selected and used for the functional annotation clustering.

### 2.6 NM-induced lung response data sets

Datasets examining differential gene expression in mouse lung exposed to nano-titanium dioxide, carbon black or carbon nanotubes (nano-TiO2, CB and CNTs) were compiled from GEO. The GEO accession numbers for these studies are presented in Table 2. These studies utilized the two-colour Agilent microarray (GPL7202 Agilent-014868 Whole Mouse Genome Microarray 4×44K G4122F and GPL10787 Agilent-028005 SurePrint G3 Mouse GE 8×60K Microarray for GSE61366) reference design [22]. The data were LOWESS normalized and probes with technical replicates were averaged. The annotation file containing the gene symbol was merged with the expression data and probes with multiple gene symbols were averaged using the median expression.

**Table 2:** NM Studies used for the Gene Set Enrichment Analysis.

Geo Accession	Nanomaterial	Doses	Time Points	Reference
GSE29042	CNT: MWCNT-7	10 µg, 20 µg, 40 µg and 80 µg	1, 3, 28 and 56 days	[23], [24], [25], [26]
GSE35193	CB: Printex 90	18 µg, 54 µg and 162 µg	1, 3 and 28 days	[27]
GSE41041	TiO2: UV-Titan L181	18 µg, 54 µg and 162 µg	1, 3 and 28 days	[28]
GSE47000	CNT: Mitsui7	18 µg, 54 µg and 162 µg	1 and 28 days	[29]
GSE60801	TiO2: NRCWE-025, NRCWE-030	18 µg, 54 µg and 162 µg	1, 3 and 28 days	[30]
GSE60801	TiO2 Sanding dust: Indoor-R, IndoornanoTiO2	18 µg, 54 µg and 162 µg	1 and 28 days	[31]
GSE60801	TiO2: Sanding dust NRCWE-032, Sanding dust NRCWE-033	18 µg, 54 µg and 162 µg	1 and 28 days	[31]
GSE60801	TiO2: NRCWE 001 (neutral), NRCWE 002 (positively charged)	18 µg, 54 µg and 162 µg	1 and 28 days	[31]
GSE61366	CNT: NRCWE-26, NM-401	18 µg, 54 µg and 162 µg	1, 3 and 28 days	[31]

### 2.7 Gene set enrichment

**Fig. 2:**
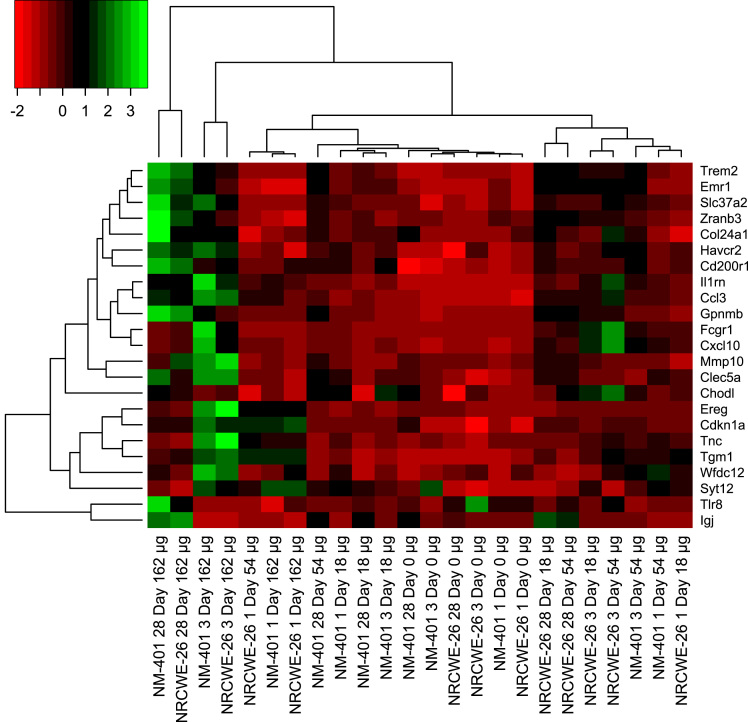
A heatmap of Bi-cluster 7 for GSE61366 is presented. Biological replicates were averaged using the median. Group medians were clustered using average linkage with the 1-correlation dissimilarity metric estimated using spearman correlations.

As the NM-induced lung response data sets contained multiple doses, the test statistic from the Attract approach was used [32]. Using this method, the overall F-statistic for the dose effect was estimated for each gene. Since large F-statistics are indicative of a strong dose effect, a bi-cluster whose distribution of F-statistics is skewed towards larger values represents an enrichment of that gene set. A two sample t-test assuming unequal variances was then conducted comparing the mean of the log2 F-statistics within the bi-cluster to the mean of the log2 F-statistics for all genes. For bi-cluster 7, a graphical representation of the group medians for GSE61366 is presented as Fig. 2. The p-values for NRCWE-26 were 0.6091, 0.0412 and < 0.0001 for days 1, 3, and 28 and similarly for NM-401 with p-values of 0.5391, 0.0005 and < 0.0001. These results were graphically reported in Williams and Halappanavar [1].

The results identified all the NMs to be inflammogenic and only CB and CNTs as potentially fibrogenic. In addition to identifying several previously defined, functionally relevant gene sets, the study also identified two novel genes associated with pulmonary fibrosis and reactive oxygen species. These results demonstrate the advantages of using a data-driven approach to identify novel, functionally related gene sets.

## Appendix A Supplementary material

1. RCode RMA normalization.R. 2. RCode Quantile normalization.R. 3. RCode LOWESS normalization.R. 4. RCode Cyclic LOWESS normalization. 5. RCode Merging biclust Bimax analysis.R. 6. RCode NMs GSEA on bi-clusters.R. 7. All Data.zip.
