# Supplementary material for: Application of bi-clustering of gene expression data and gene set enrichment analysis methods to identify potentially disease causing nanomaterials
Source: Data Brief. 2017 Oct 26;15:933–40. doi: 10.1016/j.dib.2017.10.060 (PMC5683856; doi:10.1016/j.dib.2017.10.060)
Supplement: Supplementary file 1 — Transparency document [file mmc1.docx]

**Conflict of Interest**

The authors have no conflict of interest to declare.
